# The Effects of Visual Cueing on Students with and without Math Learning Difficulties in Online Problem Solving: Evidence from Eye Movement

**DOI:** 10.3390/bs13110927

**Published:** 2023-11-14

**Authors:** Shuang Wei, Qingli Lei, Yingjie Chen, Yan Ping Xin

**Affiliations:** 1 Department of Visual Communication Design, Jiangnan University, Wuxi 214122, China; 2 Department of Special Education, University of Illinois at Chicago, Chicago, IL 60607, USA; qlei@uic.edu; 3 Department of Computer Graphics Technology, Purdue University, West Lafayette, IN 47907, USA; victorchen@purdue.edu; 4 Department of Educational Studies, Purdue University, West Lafayette, IN 47907, USA; yxin@purdue.edu

**Keywords:** visual cueing, word problem solving, mathematical learning difficulties

## Abstract

This study investigated the impact of visual cueing on attention guidance, deep-thinking promotion, and performance optimization in arithmetic word problem solving for students with mathematical learning difficulties (MLD). The participants included eight students with MLD and twenty students without MLD who attempted to solve mathematical word problems with and without visual cueing. Eye movements were recorded during the tasks. A repeated-measure design and nonparametric tests were applied to enhance the statistical power of the study. The data analysis results indicated that visual cueing effectively guided and sustained the attention of students with MLD, reducing their off-task duration. However, it showed limited influence in facilitating deep thinking and performance improvement for these students. There were no significant attention-guidance or performance-improvement effects observed in the problem-solving processes of students without MLD, who initially demonstrated better concentration levels and performance. The potential explanations for these findings are further discussed in this paper.

## 1. Introduction

Visual cues are extensively employed in the realm of education, ranging from color-coded reading materials to highlighted multimedia content [1]. People commonly use visual cues with the presumption that they positively influence students’ learning. However, a critical question arises: is this presumption supported by evidence? Researchers have been exploring the effects of visual cues on learning for several decades, expanding their exploration to encompass various learning environments along with the advancement of learning technology—from paper books to multimedia material and from online education to virtual-reality education. Their findings suggested that visual cues effectively direct students’ attention and facilitate their information-searching processes [2,3]. However, despite these promising results, it is essential to acknowledge that there is still no consensus on whether visual cues truly lead to a significant improvement in student performance.

In some studies, students achieved better performance, which was explained by the impact of visual cues in guiding students’ attention away from irrelevant areas and saving their working memory for the key information, consequently leading to better learning results [4]. However, in some other studies, students only scored well in retention tasks. In transfer tasks, students’ performance was not better [5,6], indicating that visual cues are only external models; students need to understand the information and build their own internal models to solve transfer tasks. As external tools, visual cues play a limited role in helping students understand, internalize, and apply information [5]. Some scholars’ studies even denied the role of visual cues in helping students to focus on information. They pointed out that students may just be guided and not really see the information [7]. Researchers suggested that many factors can affect the effectiveness of visual cueing, such as learning content, student groups, and the learning environment [8,9,10]. Currently, there are many visual-cueing studies available that have used different learning contents and environments, but most of them have targeted the general population. Little attention has been given to specific student groups, especially those with learning difficulties. Therefore, further in-depth research is needed to investigate the effects of visual cueing on different student groups, taking into account the unique needs and challenges faced by students with diverse learning abilities.

In this study, the effects of visual cueing on students with and without mathematical learning difficulties are explored. Student performance is one measure to evaluate the effect of visual cueing. In addition to that, it is argued that students’ eye movement, as a process-related measure, can directly reflect students’ perceptual and cognitive characteristics.

### 1.1. Visual Cueing in Online Learning

Visual cueing has been widely utilized in the practice of online learning. Visual cues like bold text, colors, arrows, or underlining are used to highlight important information in online materials [11,12]. Online multimedia resources like video may use visual cues like blinking, highlighting, or zooming in on important details [13,14]. The effects of visual cueing have been extensively investigated across various aspects, including different learning subjects (e.g., science, reading, problem solving, etc.) and diverse student groups (e.g., children, university students, low-performing students, etc.). Numerous studies have demonstrated the attention-cueing effect of visual cues [15,16]. For example, Jamet [6] employed an eye-tracking method to explore the impact of visual cues on students’ problem-solving processes and found that visual cues effectively redirected students’ attention toward relevant information while reducing their focus on unrelated areas. This allowed students to allocate more working memory to process the relevant information, leading to improved performance in retention tasks. However, the effect on transfer tasks was not significant. Moreover, the use of visual cues has been associated with a reduction in off-task behaviors in many studies, as students’ attention is effectively directed to the related information area. A study conducted by Kercood et al. [9] implemented text highlighting and observed that it increased problem-solving time for females at risk for ADHD, decreased off-task duration, and improved the females’ problem-solving performance. In addition to attention cueing and off-task duration decrease, visual cueing has demonstrated a third important effect—information-searching. In a study by Ozcelik et al. [4], color was employed in multimedia learning, resulting in an increased performance of participants in both retention and transfer tasks. They analyzed students’ eye movements during problem-solving processes and concluded that the use of color coding enhanced learning by facilitating the efficient location of corresponding information in illustrations and text.

Content-independent visual cueing has been studied, and three positive effects have been consistently verified, on attention cueing, off-task reduction, and information searching. However, beyond these effects, the role of visual cueing in facilitating understanding and improving performance remains a contentious topic. While some of the mentioned studies demonstrated that students achieved better performance with the help of visual cueing, not all research supports this finding. For example, Yeari et al. [3] conducted a study wherein text highlighting guided students’ attention to the central text area, but no significant difference in students’ processing and recall of central information was observed across the highlighted conditions. Cojean and Jamet [5] also verified the effect of visual cueing on information seeking, but they did not find any promotion of information understanding. They suggested that visual cues act as external cues, guiding students to the information but not necessarily ensuring its internalization. Similarly, Yang [13] reached a similar conclusion, noting that visual cueing effectively guides attention but may not optimize conceptual understanding or lead to improved performance. Furthermore, they have found evidence of a learning-interference effect of visual cueing on high-performing students, suggesting that for certain groups of learners, visual cueing may not be as beneficial and could potentially hinder their learning processes.

The impact of visual cueing on subject learning has been explored in various studies, encompassing multimedia learning, science learning, and text reading. Some research has explored its effect on different participant groups [17]. However, the research results in this area are mixed, and the underlying reasons for these inconsistencies remain ambiguous. In light of this, the current study aims to examine the effect of visual cueing in mathematical word problem solving. The researchers sought to determine if any differences exist in the effects of visual cueing between students with and without MLD and analyze the underlying factors contributing to these differences. To achieve this, the researchers employed eye-tracking technology, aiming to provide empirical evidence regarding the impact of visual cueing and enhance our understanding of the problem-solving characteristics of two student groups.

### 1.2. Eye-Tracking Technology in Education

The application of eye-tracking technology in educational settings dates back to the 1980s and 1990s, when researchers began using eye-tracking data to understand and analyze learners’ comprehension processes when dealing with textual and pictorial information [18,19,20]. Van Gog and Scheiter [21] emphasized that eye-tracking data can provide valuable insights into learners’ visual behaviors, revealing what they are looking at, how long they focus on specific elements, and the sequences of their gazes’ movements across different representations. Eye trackers measure various eye-movement parameters, such as fixation duration, gaze duration, and transition frequency, to capture these behaviors objectively (for a comprehensive list of eye-movement metrics, please refer to the paper by Bednarik et al. [22]).

Researchers have used eye-tracking technology to investigate various aspects of learning behavior. For example, Brunyé and Taylor [23] explored how different learning goals may impact learners’ outcomes. Schwonke et al. [24] observed changes in attentional behaviors when learners interacted with different representations and how these behaviors influenced learning. Eye-movement data have been deemed more objective and reliable than subjective self-reports [25].

Beyond attention, researchers have attempted to connect eye movements with other learning behaviors. Just and Carpenter [19] proposed the eye–mind hypothesis, suggesting that the eyes fixate on the areas where the mind is actively engaged. She and Chen [26] compared the effects of different multimedia methods on students’ eye fixation behaviors and empirically verified a direct correlation between the duration of eye fixations and the depth of learning. Wu et al. [27] adopted eye-movement measures, such as total fixation duration, number of long fixations, and pupil size, to predict participants’ performance. Susac et al. [25] analyzed students’ eye fixations, reaction time, performance, and questionnaires to reveal their strategies in equation solving. Eye-tracking technology has been widely applied in online-learning research and is recognized as a powerful tool to study student learning behavior. In our study, we utilized an eye-tracking system to analyze the effect of visual cueing on students’ problem-solving processes. Alongside eye-movement data, we collected student problem-solving processing data and recorded problem-solving videos to test the following hypotheses:

**H1:**  *Off-task behavior. We hypothesize that students with MLD exhibit more off-task behavior than students without MLD, which can be indicated by their off-task duration (H1a). With the help of visual cueing, the off-task behavior of students with MLD is expected to decrease significantly (H1b).*

**H2:**  *Attention guidance. We hypothesize that the attention of students with MLD is less concentrated than those without MLD, which can be reflected in their fixation durations on different areas of the screen (H2a). However, visual cueing can guide the attention of students with MLD to the cued area and help maintain their attention (H2b). Visual cues may also have a guiding effect on students without MLD, although the effect may not be as significant as in the MLD group (H2c).*

**H3:**  *Deep thinking. We hypothesize that visual cueing can encourage students both with and without MLD to think more deeply about the cued area and process the corresponding information better.*

**H4:**  *Performance. We hypothesize that visual cues can improve the problem-solving performance of students both with and without MLD. That is, in the cueing condition, students’ performance is expected to be better than their performance in the no-cueing condition.*

## 2. Materials and Methods

**Figure 1:**
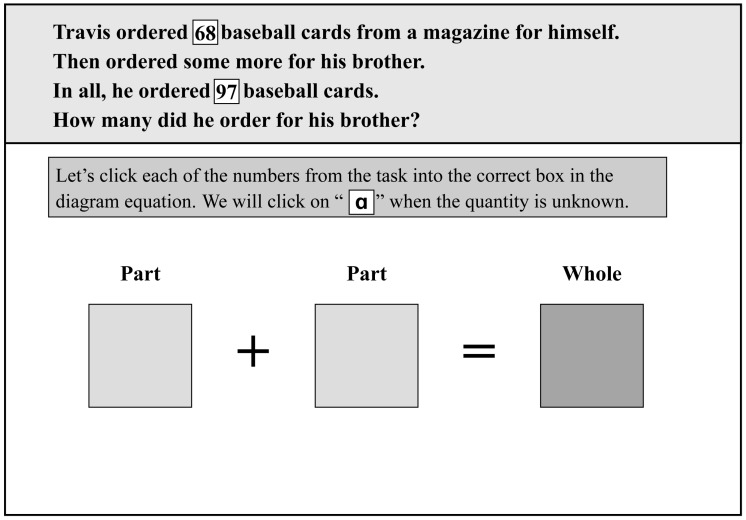
A sample screenshot of the mathematics problem-solving tasks (COMPS-RtI Tutor© [30]) Numbers and the letter ‘*a*’ in the problem are made as tags, allowing students to click on them and put them into equation boxes to complete the equation.

The experiment’s tasks were taken from a Conceptual Model-Based Problem-Solving (COMPS) tutor [28], which we developed to promote the additive mathematics problem-solving performance of students with MLD. The tasks were formulated using text at a second-grade reading level [29] and were specifically designed to enhance students’ understanding of real-world problems and improve their abilities to create algebraic equations from word problems. Figure 1 illustrates an example of the mathematical word problems that the students encountered. In this problem, students were asked to apply numbers in the problem to the diagram equation and use an unknown quantity, “a”, to complete the equation. The problem-solving process was divided into three segments. First, the COMPS tutor read the question content to the students (reading segment); then, a voice prompt was played to decompose the question content and provide hints regarding the association between the question content and the algebraic equation (instruction segment). Subsequently, students executed the necessary operations to complete the equation (operation segment).

### 2.1. Participants

Participants were 2nd- or 3rd-grade elementary school students from a midwestern state of the United States. They were recruited based on teacher referrals, which specifically targeted students who experienced significant challenges in mathematics word problems. For those students whose parents granted permission, the Stanford Achievement Test [31] Problem-Solving Subtest was administered to assess their mathematics proficiency. Students whose scores fell below the 35th percentile were considered to have MLD [32]. These students then took a word problem-solving criterion test [28] to determine whether they had difficulties in solving targeted word problems. Students with scores below 60% on the criterion test were selected as participants for this study. Based on their test results, eight students with MLD and twenty students without MLD were recruited to participate in the study. Table 1 presents participant demographic characteristics.

### 2.2. Experiment Design

We utilized a two-group within-subject experimental design. Students with MLD were assigned to the experimental group, while students without MLD were assigned to the control group. Both groups of students attempted four word problem-solving tasks in random order. Two tasks had instruction segments with visual cues (text blinking and coloring), while the other two tasks had no visual cues. The combinations of the task and the visual cues were randomized. Each task and the visual cues were presented to each participant only once.

The study was conducted in the computer labs of schools during multiple sessions. The computer labs were designed with similar environmental settings, illuminated by both natural and artificial light, ensuring a minimum illumination level of 50 foot-candles [33]. All students used the same computer models—Dell Precision 3520 workstations with 15.6-inch displays and a resolution of 1920 × 1080 pixels. A Tobii Pro X3-120 eye tracker (120 HZ) was mounted at the bottom of each laptop screen to record students’ eye movements and interactive behavior. The Tobii Pro X3-120 eye tracker is extra slim (115 × 111 × 32.7 mm), minimizing interaction interference and attention distraction for the participants. Furthermore, the eye tracker can capture students’ eyes within a distance of 50 cm to 90 cm [34]. To ensure the quality of eye tracking, the distance between the eye tracker and the student was approximately 65 cm. Both groups of students received exactly the same treatment process. After students were seated in front of computers, they were informed that they would be working on a tutor session and completing four word problem-solving tasks, which might take thirty minutes. The students were encouraged to do their best and assured that the study was not an exam, and their performance would not be judged. Once the eye-tracking system was calibrated, students took control of the computers and started their tasks. While students were performing tasks, researchers did not sit beside the students or remain too close unless students asked for help. This approach aimed to minimize pressure on the students and encourage more natural behavior during the tasks.

### 2.3. Measures

The research collected two types of data: processing data and eye-movement data. Processing data included log data and student performance data recorded by the COMPS program. Student eye-movement data included fixation, saccade, and other related measurements. The eye-tracking equipment, Tobii Pro X3-120, collected students’ eye-movement data, which was stored in its bundled software—Tobii Pro Studio 3.4.8 (Reston, VA, USA). This software allowed researchers to observe participants’ eye movements in real time, define areas of interest (AOIs), and export the eye-movement dataset as Excel files. The eye-movement data, such as fixation positions in X and Y directions (in pixels), timestamp, fixation duration (in milliseconds), fixation count, and saccade count, were recorded.

The measures adopted in the research included the off-task duration ratio (ODR) of a segment, the fixation duration ratio (FDR) on AOIs, and the long fixation duration ratio (LFDR) on AOIs. The decision to use duration ratio instead of absolute duration was due to the problem-solving process being divided into three segments (reading, instruction, and operation) of varying lengths. To ensure comparability across segments, the absolute durations were divided by the respective segment durations.

Students’ eye-movement data from Tobii Studio were imported into a MySQL database. Additionally, processing data, including the start and end times of each segment (in milliseconds), were stored in the database. The task layout was defined as areas of interest (AOIs) including question area, equation area, and white area. The coordinates of the question area and equation area (in pixels) were recorded. The white area was obtained by subtracting the question and equation area from the entire area. The calculation methods for the measures are provided below.

#### 2.3.1. Off-Task Duration Ratio (ODR)

We analyzed students’ eye-movement data to identify periods without any fixations and reviewed the video recordings of problem-solving sessions to detect any off-task behavior, such as chatting, staring into space, or resting their heads on the desk [35,36]. The duration of off-task behavior was subsequently divided by the corresponding segment duration to obtain the ODRs for both the reading and instruction segments.

#### 2.3.2. Fixation Duration Ratio (FDR)

The problem-solving process was divided into reading, instruction, and operation segments, with layout defined by question, equation, and white area. The FDR was calculated by dividing a student’s fixation duration in an area during a segment by the segment duration. Analyzing FDRs helps us recognize students’ fixation distribution and their shifting focus during the problem-solving processes.

#### 2.3.3. Long Fixation Duration Ratio (LFDR)

A fixation lasting longer than 500 ms is considered a long fixation [27]. Researchers believe that long fixations indicate deep thinking [26]. Therefore, LFDR is an important measure for the exploration of differences in information processing and problem-solving patterns between students with and without MLD. We calculated LFDRs by dividing the total duration of long fixations in a specific area during a segment by the duration of that segment.

## 3. Results

A counterbalanced design was utilized to ensure that no control variables (task order, visual cues) significantly differed among the experiment conditions. Due to the small sample sizes in the two groups, non-parametric tests were employed to analyze the significance of group differences. The Mann–Whitney U test was used to explore differences between the two student groups (two independent sample tests), while the Wilcoxon test was applied to examine differences between the cueing and no-cueing conditions within the same student group (two related sample tests). The data analysis was conducted using IBM SPSS Statistics 23 (Armonk, NY, USA). The eta-squared (η^2^) was reported as a measure of non-parametric tests, where effect sizes of 0.01, 0.06, and 0.14 correspond to small, medium, and large effect sizes, respectively [37].

### 3.1. Off-Task Duration Ratio (ODR)

**Figure 2:**
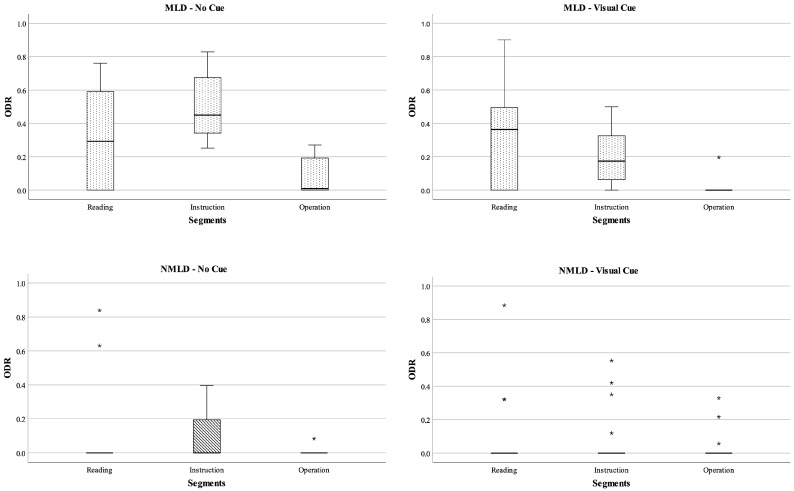
Medians and interquartile ranges (IQRs) for the ODRs of the MLD and NMLD groups. Note: * represent outliers.

Figure 2 displays the descriptive statistics for the ODRs of the two groups. The ODRs of the MLD group are significantly larger than the non-MLD group (NMLD) in the reading segments, which had no visual cueing (U = 29.50, *p* = 0.01, η^2^ = 0.24). This result supports H1a, indicating that students with MLD exhibit more off-task behavior than students without MLD.

In the instruction segment of the non-visual-cues condition, the ODR median of the MLD group increased from 0.29 in the reading segment to 0.45, whereas the NMLD group showed little change. This led to a significant difference in ODR between the two groups in the instruction segment (MLD—med = 0.45, NMLD—med = 0.00, U = 5.00, *p* = 0.00, η^2^ = 0.52). However, in the visual-cueing condition, the ODR median of the MLD group decreased from 0.45 to 0.17. The Wilcoxon test revealed a significant decrease in the ODRs of the MLD group in the cueing condition compared to the no-cueing condition (z = −2.38, *p* = 0.02, η^2^ = 0.20). This indicates that visual cues effectively reduced the off-task duration for the MLD group (H1b). In the operation segment, both the MLD- and NMLD-group students were more focused on solving the problems and were less likely to be off-task.

In conclusion, the ODR of the MLD group was greater than that of the NMLD group. The ODRs of students without MLD remained relatively stable, with little effect from segments or cueing conditions. In contrast, the ODR of the MLD group decreased when visual cues were applied; otherwise, it increased significantly over time.

### 3.2. Fixation Duration Ratio (FDR)

Table 2 presents the descriptive statistics for the FDRs of two student groups under different conditions. We analyzed student eye fixation duration and location changes over time, specifically focusing on the FDR differences between the MLD and NMLD groups in the no-cueing conditions. Additionally, we compared the FDR differences between the cueing and no-cueing conditions for both student groups.

#### 3.2.1. Effects of Student Groups on the FDR in the No-Cueing Condition

Most student fixations were in the question area, which is the primary information area. When there was no visual cueing, the FDR median of students with MLD was 0.30 in the reading segment, and then it decreased to 0.20, while the FDR of students without MLD was 0.45, and then it decreased to 0.31 over time.

In the equation area, fewer fixations were observed compared to the question area in both the reading and instruction segments. The FDR median of the MLD group in the equation area was 0.01. In contrast, the FDR of the NMLD group in the equation area increased in the instruction segment, indicating that the students without MLD expanded their focus to seek more information related to the problem. This resulted in a significant FDR difference in the equation area between the two student groups in the instruction segment (MLD—med = 0.01, NMLD—med = 0.05, U = 29.50, *p* = 0.01, η^2^ = 0.24).

Compared to the equation area, the MLD group exhibited greater attention toward the white area. Their FDR median on the white area in the reading segment was 0.02, which subsequently increased to 0.03. An analysis of FDRs on the whole area shows that the MLD group’s fixation duration decreased in the instruction segment and was significantly lower than that of the NMLD group (MLD—med = 0.23, NMLD—med = 0.56, U = 30.00, *p* = 0.01, η^2^ = 0.23). This suggests that the MLD group displayed reduced concentration and shifted their attention away from the screens.

Overall, students with MLD demonstrated less concentration compared to students without MLD, and their attention became more scattered over time (H2a). As a result, their FDR significantly decreased in the instruction segment. Conversely, students without MLD also showed a diversion of attention in the instruction segment. However, unlike MLD students, who tended to become off-task or focus on elements other than the task, students without MLD started to notice and seek new related information, specifically focusing on the equation.

#### 3.2.2. Effect of Visual Cueing on the FDRs of Students with and without MLD

Wilcoxon tests were conducted to analyze the FDR difference between the cueing and no-cueing conditions. In the no-cueing condition, the FDRs for question and whole areas of interest decreased for the MLD group throughout the reading and instruction segments. However, in the condition with visual cueing, this decreasing trend was mitigated to some extent. The FDR of the instruction segment for the question area was significantly higher in the cueing condition compared to the no-cueing condition (z = −2.52, *p* = 0.01, η^2^ = 0.23). This indicates that visual cues can enhance the attention of students with MLD toward the screen, especially in the cued area (H2b).

However, the increasing effect of visual cues did not show in the NMLD group. Students’ FDR in the cued area in the cueing condition was not significantly higher than in the no-cueing condition. This result is opposite to our hypothesis (H2c). Even worse, on the equation area, the FDR in the cueing condition was significantly lower than in the no-cueing condition in the instruction segment (z = −2.70, *p* = 0.01, η^2^ = 0.26). This suggests that students without MLD reduced their exploration of the related information. In conclusion, visual cueing can efficiently increase the fixations of students with MLD in the cued area and prevent their distraction. With the help of visual cueing, the FDRs of the MLD group can be improved to a similar level as the NMLD group. But visual cueing has little effect on the attention guidance and maintenance of students without MLD and may even decrease their information exploration attempts.

### 3.3. Long Fixation Duration Ratio (LFDR)

Fixation duration is believed to reflect ongoing cognitive processes. Consequently, long fixations lasting longer than 500 ms are often regarded as indicative of deeper cognitive engagement [27]. In this study, we examined the LFDR of students both with and without MLD to gain insights into their cognitive processes and the impact of visual cueing on their information processing.

#### 3.3.1. Effect of Student Groups on LFDR in the No-Cueing Condition

In the reading and instruction segments without visual cueing, the LFDRs of the MLD group were consistently lower than those of the NMLD group in all areas except the white area. Particularly in the instruction segment, the LFDR medians of the MLD group were 0.01 and 0.00 on the question and equation areas, respectively, while the LFDR medians for the NMLD group were 0.07 and 0.01, respectively. These findings suggest that students with MLD may not engage with the problem and the equation as deeply as students without MLD. In contrast, students without MLD seem to initiate deeper cognitive processing early in the problem-solving tasks. Table 3 presents the descriptive statistics for the LFDRs of two student groups under different conditions.

#### 3.3.2. Effect of Visual Cueing on LFDRs of Students with and without MLD

Upon the introduction of visual cueing in the instruction segment, the LFDR median of the MLD group in the cued area increased substantially from 0.01 to 0.10, although this increase did not reach statistical significance. Furthermore, no significant changes in the LFDR were observed in other areas of the screen. These results suggest that visual cueing may have a limited impact on the fostering of deeper thinking among students without MLD.

Similarly, no positive effect of visual cueing on the LFDRs of the NMLD group was observed. Moreover, in the equation area, the LFDR in the cueing condition was significantly lower than in the no-cueing condition (z = −2.70, *p* = 0.01, η^2^ = 0.26). One possible explanation for this finding is that students without MLD were primarily focused on the question area where visual cueing was applied. As a result, they may have allocated less cognitive energy to processing peripheral information in the equation area, leading to a decrease in their long fixations in that area. Overall, the results suggest that visual cueing does not significantly enhance LFDRs for students both with and without MLD. Therefore, Hypothesis 3, proposing that visual cueing promotes the occurrence of long fixations indicative of deep engagement, is not supported.

This indicates that visual cueing may not be effective in encouraging deeper cognitive processing, as long fixations are considered to indicate deep processing in completing a problem-solving task [38].

### 3.4. Performance

The descriptive statistics of the MLD and NMLD groups’ performance are displayed in Table 4. In the no-cueing condition, the performance of students without MLD appears to be slightly better than that of students with MLD. Two Wilcoxon tests were conducted to examine performance differences between the cueing and no-cueing conditions within each student group. The results indicated no significant performance difference for students with MLD. However, for students without MLD, their performance in the cueing condition was significantly lower than in the no-cueing condition (z = −2.53, *p* = 0.01, η^2^ = 0.23). This suggests that students without MLD generally exhibited better problem-solving performance in the no-cueing condition, which contradicts Hypothesis 4.

## 4. Discussion

The off-task duration of students in problem-solving tasks serves as an indicator of their engagement to some extent [9]. In the reading segment, students with MLD displayed more off-task behavior compared to students without MLD. Over time, students with MLD exhibited a diminishing interest in the task and became increasingly distracted from the screen, as indicated by the notable increase in their off-task duration in the instruction segment. Visual cueing efficiently reduced their off-task duration and maintained their attention on the screen, aligning with the findings from Kercood et al. [9] indicating that visual cues reduce students’ off-task behavior and enhance engagement in problem-solving processes. However, according to the data analysis results, visual cues had little effect on reducing the off-task duration of students without MLD, as they demonstrated a high level of attentiveness to the tasks and exhibited minimal off-task behavior, regardless of whether cueing was present or not in the experiment conditions.

Based on the eye–mind assumption of Just and Carpenter [19], the analysis of students’ fixations provides insights into their attentional distribution on the screen. In the no-cueing condition, students with MLD exhibited smaller fixation duration ratios on the question and equation areas compared to students without MLD. However, they displayed larger fixation duration ratios on the white area, suggesting that students with MLD allocated less attention to problem-solving-related areas. In the cueing condition, students with MLD showed increased fixation ratios on the cued area, confirming the attention-guiding effect of visual cues [7]. Despite this increase in fixation ratios, the problem-solving performance of students with MLD did not improve in the cueing condition compared to the no-cueing condition, which contrasts with the findings of Park [1]. This inconsistency can be explained by Yang’s [13] theory, which suggests that visual cueing may guide students’ attention to the cued area containing keywords but not necessarily to the key information itself. In the case of students with MLD, they might observe the cued area without engaging in deep thinking. Additionally, their fixation duration ratios for the non-cued area did not decrease. Available evidence does not support the notion that visual cueing promotes deep thinking or enhances problem-solving performance in students with MLD.

Unlike students with MLD, the use of visual cues did not lead to increased attention focus on the cued area for students without MLD. Furthermore, students without MLD showed a significant decrease in fixations on the non-cued equation area, which is consistent with the findings of Yeari et al. [3], indicating that the cueing of central information decreased the reading of peripheral information. Their LFDR on the equation area also significantly dropped, which is considered a sign of deliberative thinking. This suggests that students without MLD did not actively engage with the equation information until later in the problem-solving process. Moreover, their performance in the cueing condition was significantly lower compared to the no-cueing condition. One possible explanation could be that, influenced by the concentration effect of visual cueing, students without MLD failed to explore and process non-cued information autonomously. Although the equation is not the central information in the instruction segment, it becomes relevant during later problem solving. Without cueing, students without MLD actively explored the screen, noticed the equation area, thought about it, and attempted to predict the relationship between the question and the equation. This process aids in the construction of a mental representation of the problem. As Wu et al. [27] stated, successful problem solvers can efficiently regulate their cognitive resources. Visual cues are merely external models. Students need to recognize the relevant information, internalize it, and build their internal models [5]. Failure to do so hinders performance in tasks requiring information transformation, as demonstrated in the experiment of Yang [13]. It appears that the problem-solving tasks were relatively easy for students without MLD, as evidenced by their high accuracy rate of up to 100% in the no-cueing condition. These students already possessed sufficient cognitive abilities focused on the question area, resulting in stable fixations on the question area in both the cueing and no-cueing conditions. However, the introduction of visual cues had a limiting effect on their attention to peripheral information, potentially impeding their ability to gather additional information. It is reasonable to believe that this limiting effect of visual cues is more pronounced among high-performance students who likely have a greater capacity to process information.

It should be noted that our research specifically focused on investigating the effects of visual cueing on students with and without MLD in online word problem solving. However, solving word problems is an inherently complex task that necessitates that students comprehend the word problem, identify key information, represent it with a mathematical model, solve it, and obtain the mathematical results [39]. Numerous factors can influence students’ problem-solving performance. Various technologies and interventions are utilized to enhance students’ problem-solving skills. Visual cueing is one of many approaches employed. In addition to that, there are several limitations to this study. Firstly, the sample size of students with MLD is relatively small, as we exhausted potential participants in the participating school. To boost the statistical power of the study, we conducted a repeated-measure design and applied nonparametric statistics. Secondly, we were unable to investigate the long-term effect of visual cueing, as the repeated-measure design required participants to participate in both cueing conditions. Future research is needed to validate the long-term effect of visual cueing with larger student groups. Lastly, the study’s focus was specifically on the effectiveness of coloring and blinking as visual cues. As a result, the generalizability of the findings to other types of cueing, such as underlining, is uncertain. However, drawing from comparable studies that use different cueing techniques, it is possible to generalize some of the findings to a broader context. Further research is needed to explore the effects of various cueing methods on student performance.

In summary, this study demonstrates that visual cueing can reduce off-task duration and guide students’ attention. However, the specific effects of visual cueing vary slightly among different student groups. For students with MLD, visual cueing reduced their off-task duration and increased their attention to the cued area, but did not decrease attention to the non-cued area. It is important to note that increased attention to the cued area does not guarantee deep thinking or engagement with the content. For students without MLD, visual cues decreased their attention to the un-cued area, but did not guide more of their attention to the cued area, as they were already highly concentrated. Additionally, it is crucial to recognize that attention cueing alone does not guarantee improved learning outcomes. The effectiveness of visual cueing in enhancing learning is influenced by many factors, such as student characteristics, problem types, and the design of visual cueing. When applying cueing techniques in computer-assisted learning programs, it is vital to consider these factors. Customizing cueing strategies to align with student characteristics and specific learning objectives can increase the likelihood of desired learning outcomes.

## Figures and Tables

**Table 1:** Participant demographic characteristics.

Group	N	Age—Mean (Year)	Age—SD	Male	Female
MLD	8	8.73	0.30	37.5%	62.5%
NMLD	20	8.50	0.41	45%	55%

**Table 2:** Medians and interquartile ranges (IQRs) for the FDRs of different groups, areas, and segments.

Group	Area	No Cue				Visual Cue			
		Reading		Instruction		Reading		Instruction	
		Med	IQR	Med	IQR	Med	IQR	Med	IQR
MLD									
	Question	0.30	0.11–0.45	0.20	0.12–0.26	0.28	0.20–0.40	0.24	0.18–0.50
	Equation	0.01	0.00–0.02	0.01	0.00–0.02	0.01	0.00–0.03	0.01	0.01–0.05
	White	0.02	0.01–0.04	0.03	0.00–0.05	0.01	0.00–0.02	0.04	0.02–0.07
	Whole	0.32	0.14–0.51	0.23	0.09–0.35	0.32	0.22–0.41	0.28	0.24–0.63
NMLD									
	Question	0.45	0.29–0.68	0.31	0.20–0.55	0.37	0.09–0.62	0.35	0.12–0.52
	Equation	0.01	0.00–0.05	0.05	0.01–0.11	0.01	0.00–0.03	0.02	0.01–0.04
	White	0.01	0.00–0.03	0.02	0.00–0.04	0.00	0.00–0.02	0.01	0.00–0.03
	Whole	0.53	0.38–0.70	0.56	0.31–0.63	0.39	0.25–0.66	0.43	0.17–0.57

**Table 3:** Medians and interquartile ranges (IQRs) for the LFDRs of MLD and NMLD groups.

Group	Area	No Cue				Visual Cue			
		Reading		Instruction		Reading		Instruction	
		Med	IQR	Med	IQR	Med	IQR	Med	IQR
MLD									
	Question	0.03	0.00–0.12	0.01	0.00–0.17	0.06	0.00–0.10	0.10	0.05–0.17
	Equation	0.00	0.00–0.00	0.00	0.00–0.00	0.00	0.00–0.00	0.00	0.00–0.01
	White	0.00	0.00–0.00	0.00	0.00–0.00	0.00	0.00–0.00	0.01	0.00–0.03
	Whole	0.03	0.00–0.16	0.02	0.00–0.17	0.06	0.00–0.10	0.10	0.06–0.20
NMLD									
	Question	0.04	0.00–0.18	0.07	0.02–0.14	0.05	0.01–0.10	0.06	0.00–0.16
	Equation	0.00	0.00–0.00	0.01	0.00–0.03	0.00	0.00–0.00	0.00	0.00–0.00
	White	0.00	0.00–0.00	0.00	0.00–0.00	0.00	0.00–0.00	0.00	0.00–0.00
	Whole	0.05	0.01–0.20	0.11	0.05–0.22	0.06	0.01–0.10	0.07	0.01–0.16

**Table 4:** Medians and interquartile ranges (IQRs) for the performance of MLD and NMLD groups.

Group	No Cue		Visual Cue		Wilcoxon Test	
	Med	IQR	Med	IQR	z	*p*
MLD	0.50	0.50–1.00	1.00	0.50–1.00	−1.00	0.317
NMLD	1.00	1.00–1.00	1.00	0.50–1.00	−2.53	0.011

## Data Availability

The data that support the findings of this study are available upon request from the corresponding author. The data are not publicly available due to confidentiality and research ethics.
